# Beta1-receptor blockade attenuates atherosclerosis progression following traumatic brain injury in apolipoprotein E deficient mice

**DOI:** 10.1371/journal.pone.0285499

**Published:** 2023-05-26

**Authors:** Jintao Wang, Jessica Venugopal, Paul Silaghi, Enming J. Su, Chiao Guo, Daniel A. Lawrence, Daniel T. Eitzman

**Affiliations:** Department of Internal Medicine, Cardiovascular Research Center, University of Michigan, Ann Arbor, Michigan, United States of America; Max Delbruck Centrum fur Molekulare Medizin Berlin Buch, GERMANY

## Abstract

Traumatic brain injury (TBI) is associated with cardiovascular mortality in humans. Enhanced sympathetic activity following TBI may contribute to accelerated atherosclerosis. The effect of beta1-adrenergic receptor blockade on atherosclerosis progression induced by TBI was studied in apolipoprotein E deficient mice. Mice were treated with metoprolol or vehicle following TBI or sham operation. Mice treated with metoprolol experienced a reduced heart rate with no difference in blood pressure. Six weeks following TBI, mice were sacrificed for analysis of atherosclerosis. Total surface area and lesion thickness, analyzed at the level of the aortic valve, was found to be increased in mice receiving TBI with vehicle treatment but this effect was ameliorated in TBI mice receiving metoprolol. No effect of metoprolol on atherosclerosis was observed in mice receiving only sham operation. In conclusion, accelerated atherosclerosis following TBI is reduced with beta-adrenergic receptor antagonism. Beta blockers may be useful to reduce vascular risk associated with TBI.

## Introduction

Traumatic brain injury (TBI) is a common cause of morbidity and mortality worldwide [1]. In addition to neurocognitive deficits, TBI is associated with other comorbidities, including cardiovascular complications. In the acute setting, cardiovascular effects of TBI include stress-related cardiomyopathy [2], arrhythmias [3], and ECG changes [4], all of which are associated with increases in catecholamines. Chronic cardiovascular effects, including increased coronary artery calcification and cardiovascular mortality have also been demonstrated [5]. Preclinical studies in mice support a causal relationship between TBI and atherosclerosis [6] which may be related to enhanced sympathetic activity. Chronic increases in sympathetic activity following TBI have been identified in studies of both rodents and humans [7–9]. Therapeutic blockade of beta-adrenergic receptors with beta-blockers have been shown to improve neurological and functional outcomes in humans following TBI [10, 11]. Beta-blockers may also be useful in reducing other comorbidities that may be associated with TBI. The current preclinical study was designed to determine whether the beta-receptor antagonist, metoprolol, would be effective in preventing the increased atherosclerosis observed following TBI in hyperlipidemic mice.

## Materials and methods

### Animals

Male apolipoprotein E–deficient (*ApoE*^−/−^) on the C57BL6/J strain background were purchased from Jackson Laboratory (Bar Harbor, Maine) at 8 weeks of age. Mice were housed under specific pathogen-free conditions in static microisolator cages with tap water ad libitum in a temperature-controlled room with a 12:12-hour light/dark cycle. At 10 weeks of age mice were started on a Western diet (TD88137, Harlan, WI) and at 14 weeks of age, mice underwent the CCI or sham procedures, with or without metoprolol. Mice receiving metoprolol were grouped in same cage as drug was supplied in water source. Metoprolol or vehicle control was administered via the drinking water at a concentration of 2 mg/mL for 6 weeks following TBI.

### Ethics statement

All animal use protocols complied with the Principle of Laboratory and Animal Care established by the National Society for Medical Research and were approved by the University of Michigan Committee on Use and Care of Animals. This study is reported in accordance with ARRIVE guidelines as set by the National Centre for the Replacement Refinement and Reduction of Animals in Research [12].

### Model of TBI

To induce TBI, male *ApoE*^−/−^ mice were anesthetized with 2% isoflurane and placed in a stereotactic frame (Kopf, Tujunga, CA, USA) as previously described [13, 14]. Briefly, a 5 mm circular craniotomy, centered between the bregma and lambda, was made and then a controlled cortical impact (CCI) was delivered to the midline at an impact speed of 3.00 m/s, tissue displacement of 1.1 mm, and impact duration of 50 ms. Following impact, the circular bone fragment from the craniotomy was glued back to the cranial window. The sham procedure was identical except for craniotomy and delivery of the CCI.

### Blood pressure measurement

Blood pressure and pulse rate were measured 3 weeks after CCI or sham operation in non-anesthetized, trained mice by tail plethysmography using the BP-2000 Blood Pressure Analysis System (Visitech System, Apex, NC) as previously described [15].

### Histological analysis

Quantification of atherosclerosis, the primary outcome, was performed as previously described [15, 16]. Briefly, mice were euthanized under pentobarbital anesthesia (i.p., 100 mg/kg), and arterial trees were perfused at physiological pressure and fixed in 10% zinc formalin. Paraffin-embedded hearts, which included aortic valves, were sectioned for lesion analysis. A series of 5 μm sections were obtained at the level of the aortic sinus and 4 cross sections were analyzed from each mouse. Sections were stained with hematoxylin and eosin for quantification of lesion area normalized by adjacent medial area of aorta to control for possible tangential sectioning [15, 17]. The lesion area was defined as the area between the endothelial cell layer and internal elastic lamina.

For plaque composition analysis, macrophages were quantified with an antibody to MAC3 (1:100, BD Biosciences, San Jose, CA) followed by detection of the biotin-conjugated secondary goat anti-rat IgG (BD Biosciences, San Jose, CA) with AEC substrate kit (Vector Laboratories, Newark, CA). Negative controls consisted of tissues handled identically to experimental samples except that the primary antibody was omitted. The detection system was streptavidin-HRP and endogenous peroxidase was quenched with hydrogen peroxide. Sections were counterstained with hematoxylin. Positive staining area was analyzed from three fields in each section and expressed as percentage of the total area. All images were analyzed by automated detection of positive stained area using Nikon MetaMorph software with observer blinded to treatment allocation.

### Measurements of plasma samples

Plasma samples were collected via terminal heart puncture in 3.2% sodium citrate (50μL/mL of blood) 6 weeks after TBI or sham operation. Total cholesterol was measured using Infinity cholesterol enzymatic-colorimetric kit (Thermo Fisher, #TR13421).

### Statistical analysis

All data are presented as mean ± standard deviation. Statistical analysis was carried out using GraphPad Prism. Shapiro-Wilk normality test was used for normal distribution testing. Results were analyzed using ANOVA with Dunnett’s post-hoc testing. Sample size was determined by power calculation based on variability of atherosclerosis previously established in this model [6]. No animals were excluded from analysis.

## Results

### Effect of TBI and metoprolol on baseline parameters in ApoE^−/−^ mice

**Fig 1:**
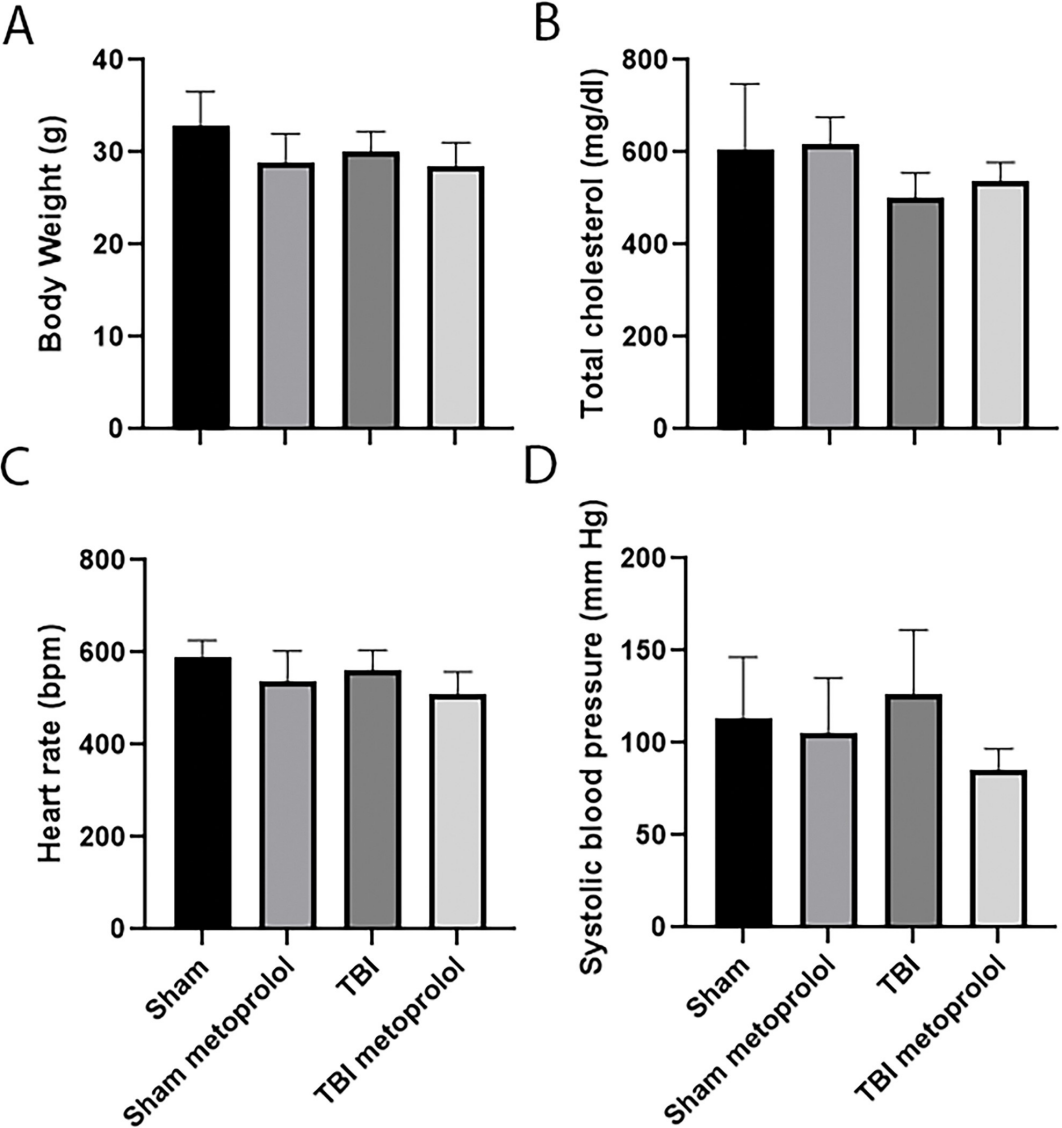
Effect of TBI and metoprolol on baseline parameters. **(A)** body weight, **(B)** total cholesterol, **(C)** heart rate, and **(D)** systolic blood pressure.

At 10 weeks of age, *ApoE*^−/−^ mice were started on a western diet to induce hyperlipidemia and accelerate the development of atherosclerosis. Four weeks later mice underwent a TBI or sham control procedure. Mice recovered quickly from the procedure and demonstrated grossly normal activity and eating behavior. Twenty-four hours following injury, mice were given metoprolol (M) or placebo (P) in the drinking water (TBI + M, n = 6; TBI +P, n = 6; Sham + M, n = 8; Sham +P, n = 9). Body weights were not different between the TBI ± M or sham groups ± M of mice 6 weeks following the procedure (Fig 1A) indicating the TBI procedure did not impair feeding or cause illness. There were also no significant differences in total cholesterol between the groups of mice (Fig 1B). Tail-cuff plethysmography was used to measure blood pressure and pulse 3 weeks following injury in non-anesthetized mice (n = 4 mice per group for this analysis). To ensure reliable and stable blood pressure measurements, mice were first trained for seven consecutive days and all blood pressure measurements were performed in the morning. No differences in pulse rate or blood pressure were observed between the groups (Fig 1C and 1D).

## Effect of TBI and metoprolol on atherosclerosis in ApoE^−/−^ mice

**Fig 2:**
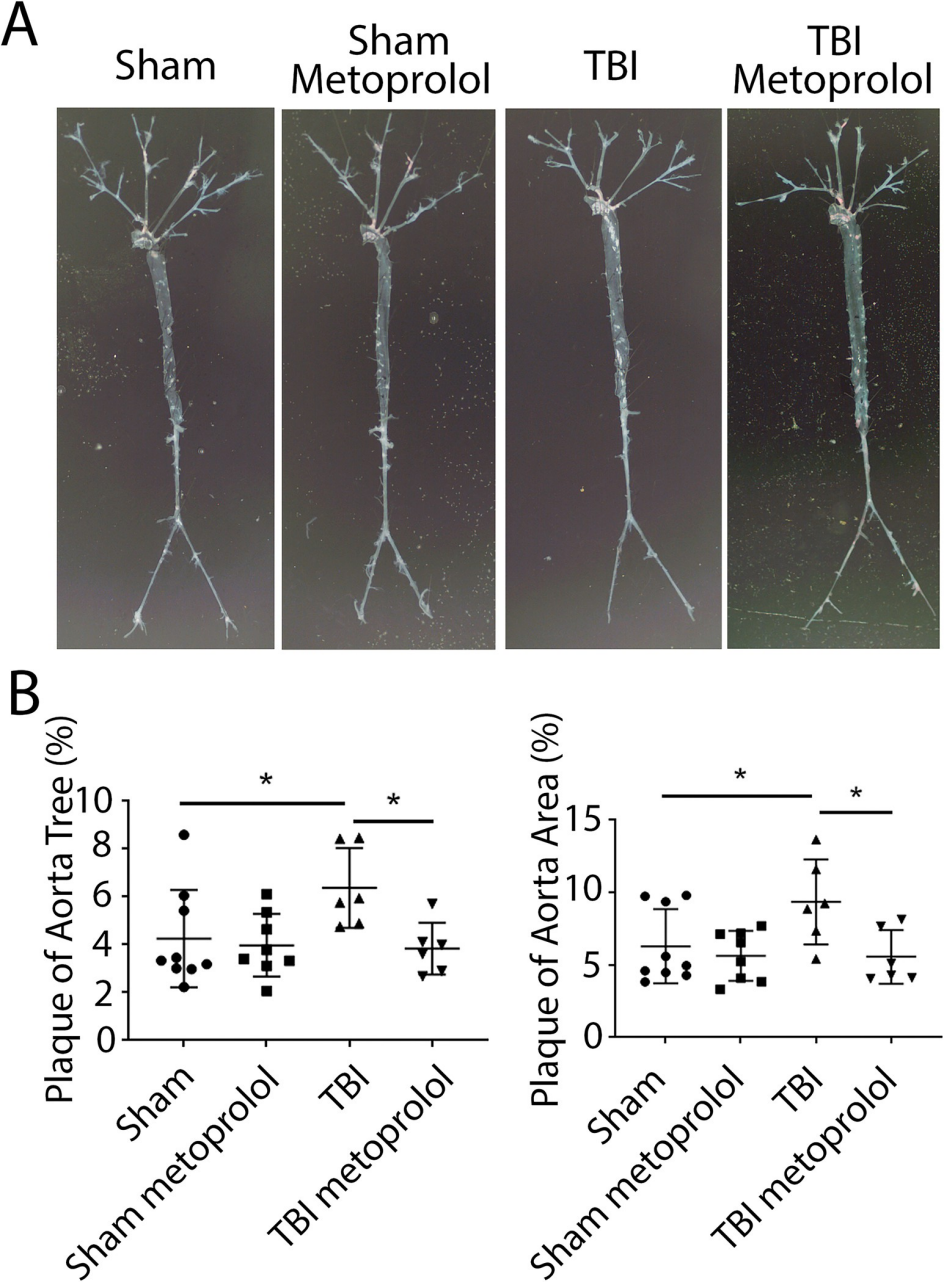
Surface area atherosclerosis involving aorta and major branches. **(A)** Representative photographs of aortic trees 6 weeks following TBI or sham operation with (+M) or without (-M) metoprolol (2 mg/mL) for 6 weeks following the surgeries. **(B)** Quantification of oil-red-O staining plaque area of aortic trees confined to the aorta or total tree 6 weeks following TBI or sham operation (*p < 0.05).

Total surface area quantitation of atherosclerosis confined to the aorta or total tree was increased in mice subjected to TBI compared to sham-operated mice (Fig 2A and 2B). However, this TBI-induced increase in atherosclerosis was attenuated in mice treated with metoprolol (Fig 2A and 2B). No effect of metoprolol on atherosclerosis was observed in sham-operated mice (Fig 2A and 2B).

**Fig 3:**
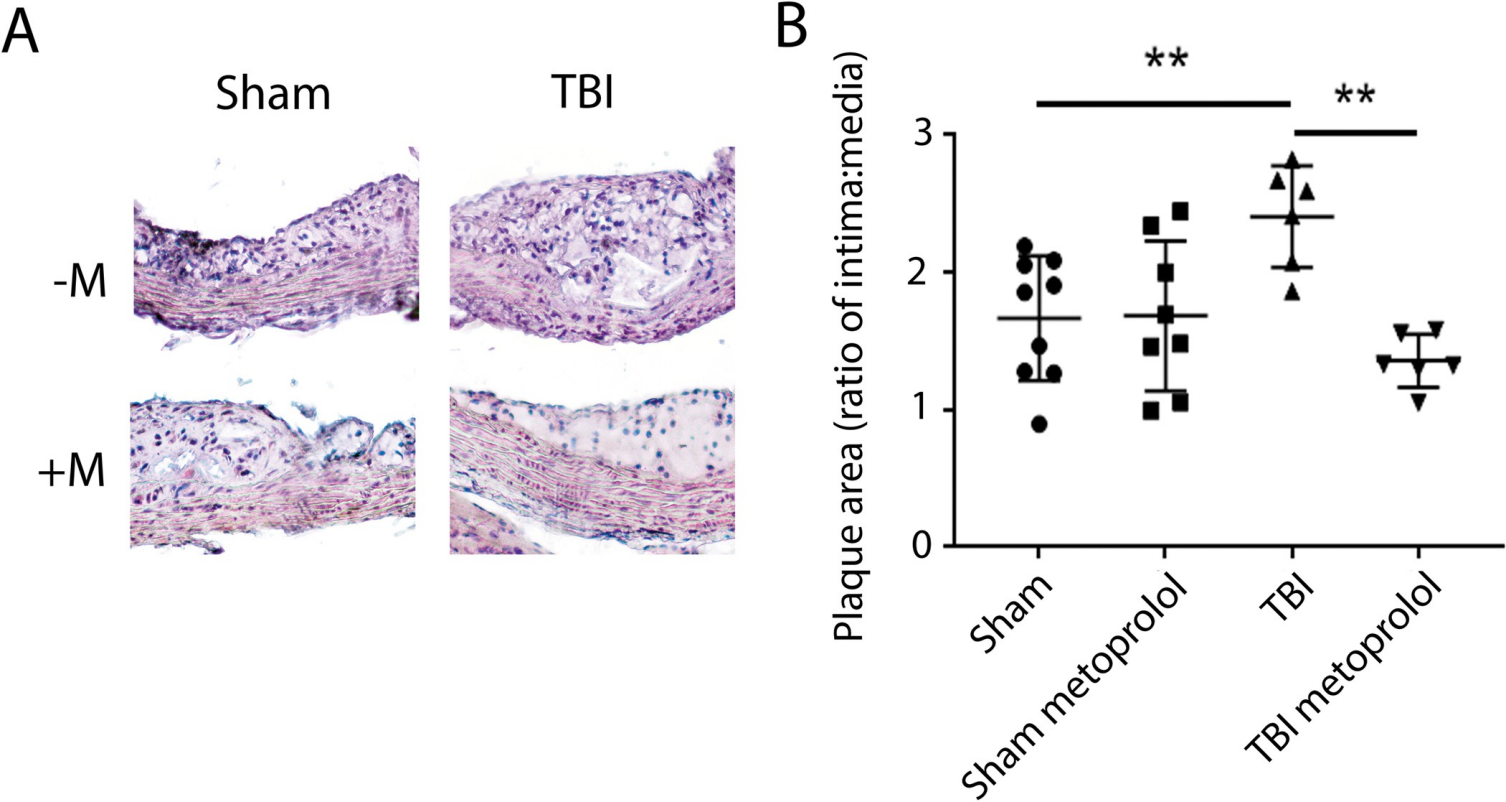
Plaque area in response to TBI and metoprolol. **A)** Representative images of sections of the aortic root stained with H and E for ApoE^-/-^ mice subjected to Sham or TBI surgeries with (+M) or without (-M) metoprolol (2 mg/mL) for 6 weeks following the surgeries. **B)** Quantification of intima:media ratio. All data are presented as mean ± standard deviation. Results were analyzed using ANOVA with Dunnetts post-hoc testing (* = p<0.05, ** = p<0.01).

Similarly, quantitation of lesion thickness performed at the level of the aortic sinuses revealed increased I/M (intima/media) ratio in mice subjected to TBI compared to sham-operated mice (Fig 3A and 3B). This TBI-induced increase in atherosclerosis was attenuated in mice treated with metoprolol (Fig 3A and 3B) while no effect of metoprolol on atherosclerosis was observed in sham-operated mice.

**Fig 4:**
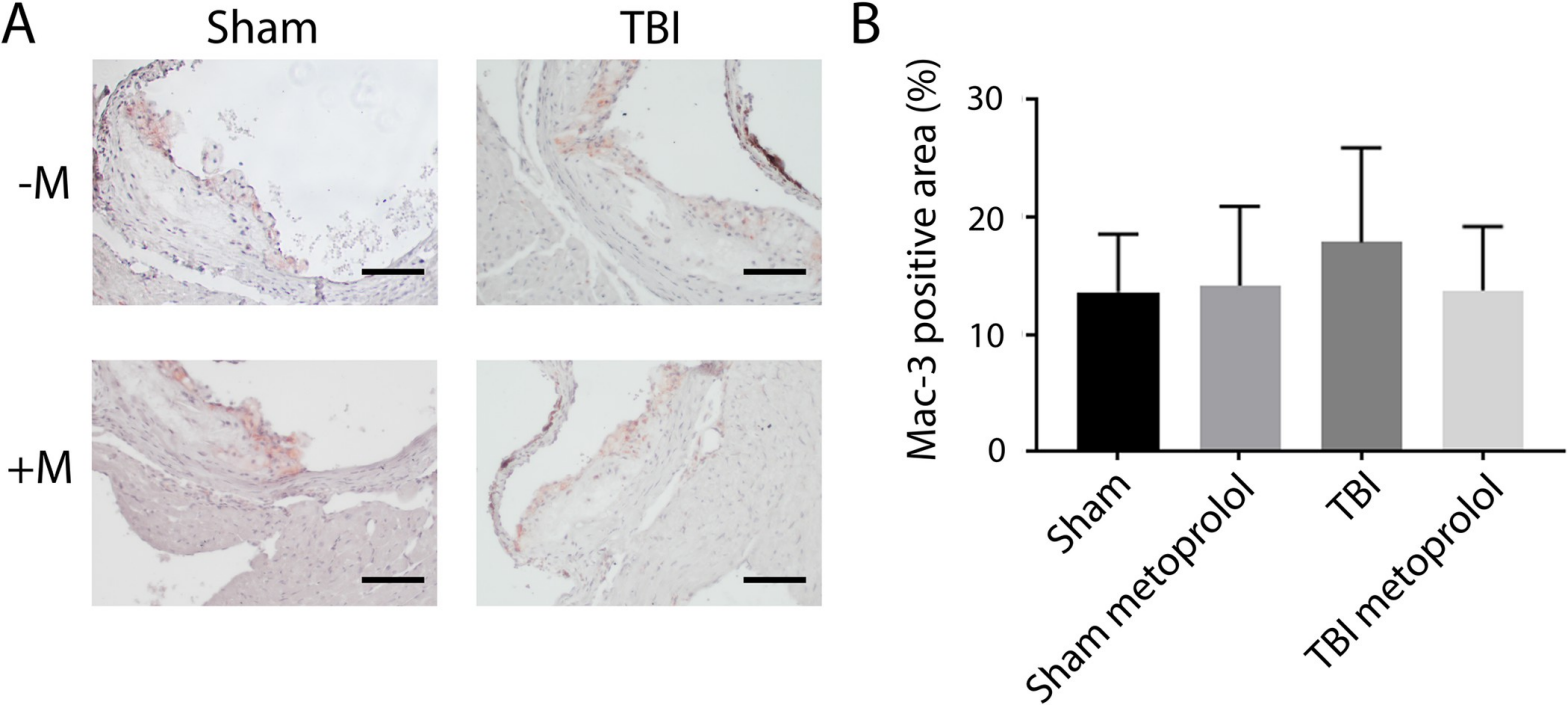
MAC3 immunostaining in response to TBI and metoprolol. A) Representative images of sections of the aortic root stained with anti-MAC3 antibody for ApoE-/- mice subjected to Sham or TBI surgeries with (+M) or without (-M) metoprolol (2 mg/mL) for 6 weeks following the surgeries. B) Quantification of MAC3 staining as percent of the plaque area. Scale bar: 50μm. All data are presented as mean ± standard deviation.

Plaque area occupied by MAC3 positive staining cells was not different in the TBI-treated mice compared to sham-treated mice and not different in TBI mice treated with metoprolol (Fig 4A and 4B).

## Discussion

Cardiovascular complications are increased following brain injury [5, 18] and these effects may be mediated by catecholamine surges as chronic and paroxysmal sympathetic hyperactivity are common after traumatic brain injury [19]. Sympathoadrenal activation following TBI has been shown to induce a coagulopathy and endotheliopathy as evidenced by biomarkers and these effects have been associated with a poor prognosis [20]. In a preclinical model, TBI was shown to accelerate atherosclerosis in hyperlipidemic mice [6].

Pharmacologic blockade of adrenergic receptors, which mediate effects of sympathetic hyperactivation, with beta blockers have been shown to improve neurologic outcome following TBI [10, 11] and a beneficial effect on survival has been demonstrated with metoprolol following severe TBI that was independent of heart rate [21]. Since a murine model demonstrating effects of TBI on a relevant vascular endpoint has been previously established [6], the current study was designed to determine whether therapy with a beta blocker might be effective in preventing TBI-induced accelerated atherosclerosis.

The CCI model has been commonly used in mice to explore pathways involved in post-traumatic brain injury [22]. As in humans, cascades are activated subsequent to injury that lead to chronic systemic effects involving apoptosis [23], inflammation [24] and oxidative stress [25, 26]. The mortality rate associated with the CCI model in rodents is low, so long term survival studies are feasible. Pathophysiological changes have been shown to occur even 1 year after CCI, including ongoing neurodegeneration, microglial activation [27], and neurologic compensatory responses [28]. The model may therefore be useful to analyze effects of TBI on vascular disease processes.

Atherosclerotic-prone rodents are widely employed to genetic and environmental factors involved in atherosclerosis [29]. Vascular endpoints are accelerated with a western diet [15, 29–31], allowing the current study to focus on a timepoint 6 weeks following the TBI.

Consistent with a previous study [6], atherosclerosis was increased in mice subjected to TBI. This effect was not associated with increases in blood pressure or pulse. Previous studies have demonstrated effects of TBI on leukocyte activation and biomarkers of endothelial adhesiveness [6], although the precise mechanism(s) for acceleration of atherosclerosis remains to be proven. In the current study, therapy with the β1-adrenergic antagonist, metoprolol, prevented the increase in atherosclerosis following TBI. This was not associated with hemodynamic effects as no difference between the groups were noted related to pulse or blood pressure. A previous study demonstrated acceleration of atherosclerosis following stroke in ApoE deficient mice suggesting common proatherogenic pathways may be activated following acute cerebrovascular events [32]. While further mechanistic studies are warranted to define the specific downstream mediators responsible for vascular effects of TBI, these findings are consistent with an important role of the central nervous system and catecholamine signaling in promoting atherogenesis. In addition to systemic catecholamine effects on circulating monocytes or endothelial cells following brain injury, efferent sympathetic peripheral nervous system axons may be stimulated to produce adrenaline locally in disease-prone arteries leading to enhanced inflammatory plaque activity [16]. The current study was limited by the use of a single, selective beta1 receptor antagonist. Additional studies are therefore necessary to also delineate the contributions of various beta receptor pools and downstream mechanism(s) responsible for beta1-blocker-mediated atheroprotection following TBI. Measurement of plasma steroid levels and catecholamines could also be revealing, in addition to detailed analysis of circulating leukocytes and plaque characteristics. However given benefits in neuroprotection and possible vascular protection, the threshold may be lowered for using this relatively benign class of drugs in patients following TBI.
